# CD44, Sonic Hedgehog, and Gli1 Expression Are Prognostic Biomarkers in Gastric Cancer Patients after Radical Resection

**DOI:** 10.1155/2016/1013045

**Published:** 2015-12-29

**Authors:** Chen Jian-Hui, Zhai Er-Tao, Chen Si-Le, Wu Hui, Wu Kai-Ming, Zhang Xin-Hua, Chen Chuang-Qi, Cai Shi-Rong, He Yu-Long

**Affiliations:** ^1^Division of Gastrointestinal Surgery Center, The First Affiliated Hospital of Sun Yat-sen University, Guangzhou 510080, China; ^2^Gastric Cancer Center, Sun Yat-sen University, Guangzhou 510080, China

## Abstract

*Aim*. CD44 and Sonic Hedgehog (Shh) signaling are important for gastric cancer (GC). However, the clinical impact, survival, and recurrence outcome of CD44, Shh, and Gli1 expressions in GC patients following radical resection have not been elucidated.* Patients and Methods*. CD44, Shh, and Gli1 protein levels were quantified by immunohistochemistry (IHC). The association between CD44, Shh, and Gli1 expression and clinicopathological features or prognosis of GC patients was determined. The biomarker risk score was calculated by the IHC staining score of CD44, Shh, and Gli1 protein.* Results*. The IHC positive staining of CD44, Shh, and Gli1 proteins was correlated with larger tumour size, worse gross type and histological type, and advanced TNM stage, which also predicted shorter overall survival (OS) and disease-free survival (DFS) after radical resection. Multivariate analysis indicated the Gli1 protein and Gli1, CD44 proteins were predictive biomarkers for OS and DFS, respectively. If biomarker risk score was taken into analysis, it was the independent prognostic factor for OS and DFS.* Conclusions*. CD44 and Shh signaling are important biomarkers for tumour aggressiveness, survival, and recurrence in GC.

## 1. Introduction

Due to an increased early detection rate and therapeutic advancements, the survival of gastric cancer (GC) patients has improved in the past 3 decades worldwide. However, GC remains the second leading cause of cancer death in China [1], mainly because of the disappointing early detection rate in China, early tumour recurrence, and high chemotherapy resistance. Hence, it is essential for gastroenterologists to identify effective biomarkers for evaluating the early detection of GC, which may also be targets for novel therapies for this deadly disease.

Cancer stem-like cells (CSCs) are defined as rare cells in malignant tumours with the ability to self-renew and to differentiate into various heterogeneous cancer cell lineages [2]. Abnormal gene expression in CSCs might be responsible for the acquisition of various genetic and epigenetic events and may play a critical role in tumour initiation, maintenance, progression, lymphatic involvement, distant metastasis, and chemoradiotherapy resistance [3]. Therefore, CSCs are considered promising tumour-specific biomarkers with potential clinical application. CD44, widely accepted as a CSCs marker for gastric cancer in many studies [4–6], is involved in cell-cell adhesion, cell-matrix interactions, and tumour metastasis [4]. However, most studies exploring the role of CD44 protein in gastric cancer included patients that received either radical resection or palliative surgery, which introduced bias into the studies. Hence, it is necessary to reevaluate the relationship between CD44 expression and clinicopathological features and long-term survival of GC patients who received radical resection. The activation of the Sonic Hedgehog (Shh) pathway affects numerous human stem cell markers in prostate [7], breast [8], and pancreatic [9] cancer. Several studies have demonstrated that increased CD44 expression activates several signalling pathways related to cancer progression and metastasis, including Shh pathway [10]. Song et al. [10] demonstrated that the Shh pathway is essential for maintenance of human gastric cancer CSCs* in vitro*. However, the clinical impact and interaction between the Shh pathway and CD44 expression in gastric cancer patients are still uncertain. Here, we aimed to find out the correlations between CD44, Gli1, and Shh expression and clinicopathological features, long-term survival, and recurrence.

## 2. Methods

### 2.1. Ethic Statement

The study was approved by the Institutional Review Board of the 1st Affiliated Hospital of Sun Yat-sen University and informed consent was obtained according to institutional regulations.

### 2.2. Clinical Samples

A total of 101 primary gastric cancer tissues were obtained at the 1st Affiliated Hospital of Sun Yat-sen University, Guangzhou, China, between January 2006 and June 2006. Patients who underwent radical gastrectomy were included, while patients who received neoadjuvant chemotherapy or chemoradiotherapy were excluded from the study. Clinicopathological parameters evaluated included age, gender, tumour location, tumour size, gross tumour type, tumour histological type, depth of invasion, lymph node involvement, distant metastasis, and TNM stage. Tumour gross types were classified as either infiltrating or noninfiltrating. Tumour histological types were classified as either well differentiated (well and moderately differentiated adenocarcinomas) or undifferentiated (poorly differentiated adenocarcinomas, signet ring cell carcinomas, and mucinous adenocarcinomas). Depth of tumour invasion, lymph node involvement, and distant metastasis were assessed according to the 7th edition of Union for International Cancer Control/American Joint Committee on Cancer (UICC/AJCC) guidelines. The potential radical resection gastric cancer patients received gastrectomy and D2 lymphadenectomy. The patients received postoperative chemotherapy using epirubicin, cisplatin, and 5-fluorouracil regimen as indicated by the concurrent UICC/AJCC guidelines. In addition, 20 human adjacent normal gastric tissues were obtained. Chronic atrophic gastritis, ulcer, and erosion were not detected microscopically in the adjacent gastric tissue samples.

### 2.3. Immunohistochemical Staining

Formalin fixed paraffin embedded human gastric cancer specimens were prepared according to the classical methods. The sections (5 *μ*m thickness) were treated with protein-blocking solution for 30 min at temperature before being incubated with primary antibodies against human CD44 (mouse monoclonal diluted 1 : 50), Shh (rabbit polyclonal diluted 1 : 100), and Gli1 (mouse polyclonal diluted 1 : 100) overnight at 4°C. All antibodies were obtained from Novus Biologicals (USA). Following incubation with the appropriate peroxidase-conjugated secondary antibody, the samples were treated with diaminobenzidine and counterstained with hematoxylin. Using bright-field microscopy, the percentage of positive cancer cells and the staining intensity was quantified independently by 2 pathologists. The mean percentage of positive tumour cells was quantified in at least 5 fields at 400x magnification and classified into one of the following 5 grades: 0 (<5% of cells had positive staining), 1 (5–25% of cells had positive staining), 2 (26–50% of cells had positive staining), 3 (51–75% of cells had positive staining), and 4 (>75% of cells had positive staining). The staining intensity of CD44, Shh, and Gli1 was scored as follows: 0 (no staining), 1 (light brown), 2 (brown), and 3 (dark brown). The percentage score and staining intensity score were multiplied to get the final staining score for each tumour specimen. The overall staining scoring system could be categorised into 2 groups: negative (0–4), positive (5–12). We defined the positive IHC staining of biomarker as biomarker overexpression.

### 2.4. Statistical Analysis

The biomarker risk score for gastric cancer in this study was the sum of the IHC score of CD44, Shh, and Gli1 proteins (positive: score 1, negative: score 0), and the patients were divided into four groups according to biomarker risk scores (groups 1–4: score 0–3). Continuous variables are presented as the mean ± SEM and categorical variables are presented as percentages (%). The two-tailed Chi-square test and Fisher's exact test for categorical variables were performed to determine statistical significance of the associations between clinicopathological parameters and the level of CD44, Shh, and Gli1 expression. Overall survival and disease-free survival rates were calculated according to the Kaplan-Meier method and were compared by log-rank tests. Cox proportional hazard models were performed for both univariate and multivariate analysis to determine prognostic significance. Spearman's rank order correlation was used to determine the correlations between the expressions of CD44, Shh, and Gli1. A *P* value of less than 0.05 was considered as statistically significant. SPSS 16.0 software (version 17.0, SPSS Inc., Chicago, IL) was used for all statistical analyses.

## 3. Results

### 3.1. Correlations between CD44, Shh, and Gli1 Expression and Clinicopathological Characteristics of Gastric Cancer

**Figure 1:**
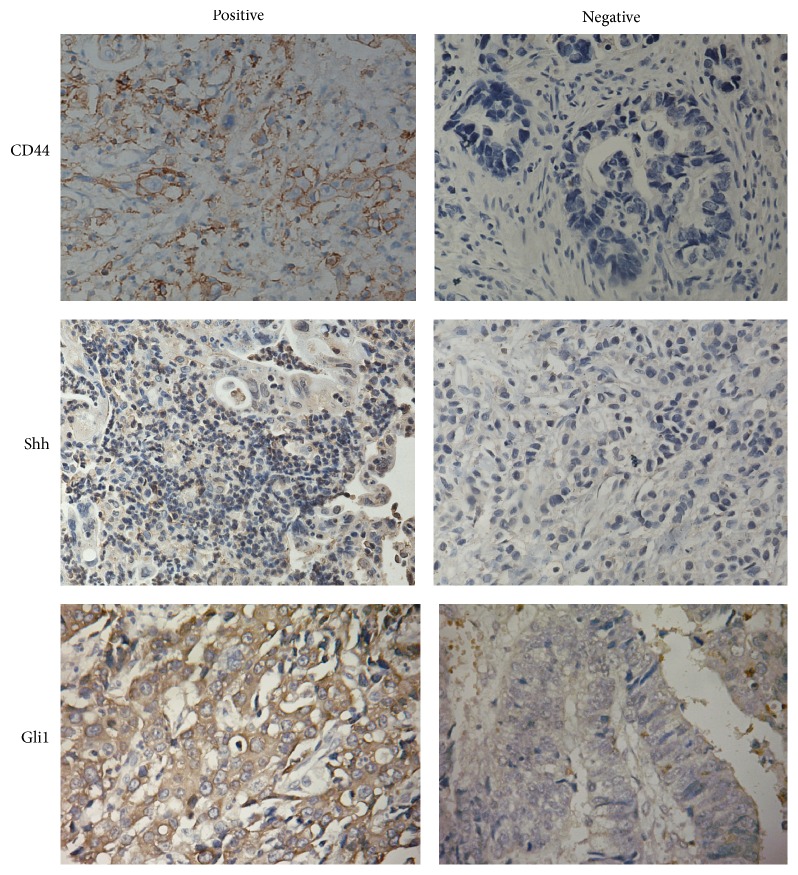
Immunohistochemical expressions of CD44, Shh, and Gli1 markers.

To investigate the role of the tumour stem cell biomarker CD44 and Shh signaling pathway in GC tumour, we evaluated the levels of CD44, Shh, and Gli1 protein in tumour tissues using immunohistochemistry (IHC) (Figure 1) and the positive stainings of Gli1, Shh, and CD44 protein were mainly localized in the nucleus, cytoplasm, and cell membrane, respectively. We found that 57.8% (59/101), 71.3% (72/101), and 57.8% (59/101) GC tumour specimens stained positively for CD44, Shh, and Gli1 protein, respectively. To further investigate the effect of CD44, Shh, and Gli1 in gastric cancer progression, we analysed the correlations between the level of CD44, Shh, and Gli1 protein and clinicopathological characteristics of GC. There were no statistically significant correlations between CD44, Shh, and Gli1 expression levels and age, gender, or tumour location (Table 1). Overexpression of CD44, Shh, and Gli1 protein was significantly associated with larger tumour size, aggressive gross type, and less differentiated tumour histological type, all of which were clinicopathological features associated with a high metastatic potential. Tumours with high CD44, Shh, and Gli1 expression had more cases of advanced tumour invasion, an increased likelihood of lymph node metastasis, advanced TNM stage (Table 1).

### 3.2. The Overexpression of CD44, Shh, and Gli1 Proteins Indicated Poor Clinical Outcome

**Figure 2:**
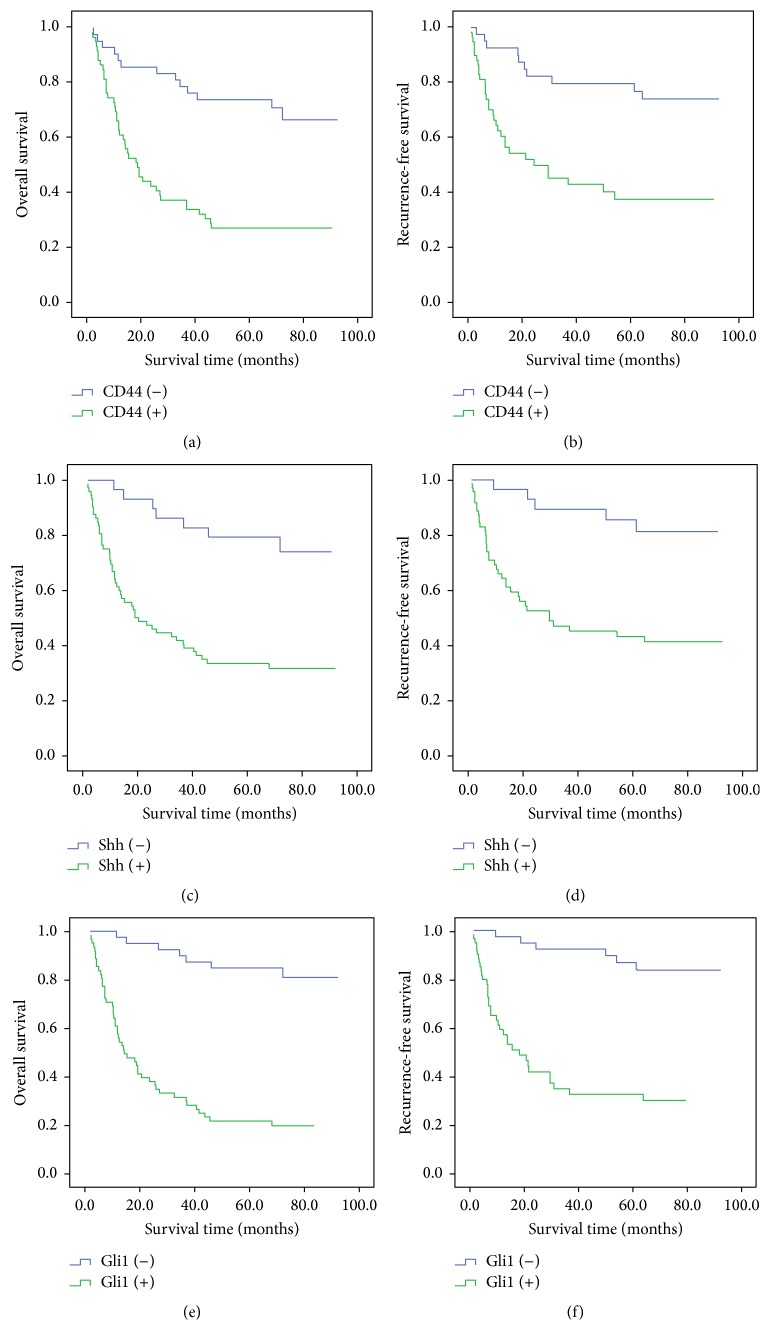
Prognostic impact of CD44, Shh, and Gli1 markers. (a) CD44 and overall survival, (b) CD44 and recurrence-free survival, (c) Shh and overall survival, (d) Shh and recurrence-free survival, (e) Gli1 and overall survival, and (f) Gli1 and recurrence-free survival.

Using Kaplan-Meier analysis and the log-rank test, we find that gastric cancer patients with CD44-positive staining had poorer overall survival. 73.8% of patients with CD44-negative tumours survived 5 years compared to only 27.1% of patients with CD44-positive tumours (Figure 2(a)) (*P* < 0.001). A similar result was observed when CD44 expression status and recurrence-free survival time were compared. The recurrence-free survival time of patients with CD44-positive tumours was lower than that of patients with CD44-negative tumours (39.0% versus 79.5%, resp., *P* = 0.001) (Figure 2(b)). Similarly, cases with Shh and Gli1 positive staining had poorer overall survival (Shh: 33.3% versus 79.3%, *P* < 0.001; Gli1: 21.3% versus 85.0%, *P* < 0.001) and recurrence-free survival (Shh: 44.6% versus 84.9%, *P* < 0.001; Gli1: 35.8% versus 86.5%, *P* < 0.001) (Figures 2(c)–2(f)).

In accordance with these results, univariate Cox regression analysis also revealed that CD44, Shh, and Gli1 status were associated with the prognosis of gastric cancer in our study (Table 2). Rather than CD44 and Shh expression levels, only TNM staging and Gli1 expression level were independent prognostic factors for overall survival of patients with GC in this study (Table 2). Similar to the results of prognostic analysis for overall survival, CD44, Shh, and Gli1 status also affected the recurrence of gastric cancer in our study (Table 3). The multivariate analysis revealed that, other than TNM stage and nodal classification, Gli1 status was the independent factor for recurrence-free survival in our study (Table 3).

### 3.3. The Correlation of CD44 Expression with the Shh Signalling Pathway in Gastric Cancer

The Shh signalling pathway regulates tumour development via cell proliferation and is involved in the progression and metastasis of a wide variety of human cancers. Hence, abnormal activation of the Shh pathway could be essential for maintenance and regulation of cancer stem-like cells in human gastric cancer. Using immunohistochemistry, we found that CD44 protein levels were correlated with those of both Shh (*r* = 0.385, *P* < 0.001) and Gli1 (*r* = 0.219, *P* = 0.028).

### 3.4. Survival Impact of Biomarker Risk Score for Gastric Cancer

**Figure 3:**
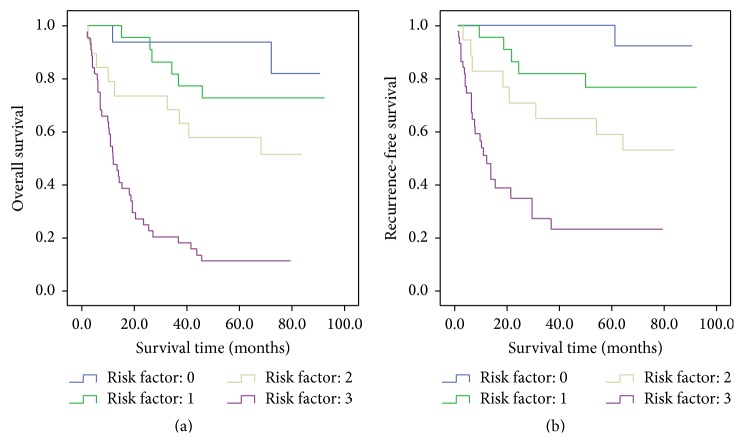
Prognostic impact of biomarker risk score system. (a) Overall survival. (b) Recurrence-free survival.

We defined the positive staining of CD44, Shh, and Gli1 proteins as score 1, and the patients were divided into four groups according to biomarker risk scores. There were prognostic differences of overall survival and recurrence-free survival among four groups (Figures 3(a) and 3(b)), and the 5-year overall survival rates and recurrence-free survival rates of biomarker risk score of 0, 1, 2, and 3 were 93.8%, 72.7%, 57.9%, and 11.4% and 100.0%, 75.6%, 61.1%, and 27.3%, respectively.

The biomarker risk score also had prognostic impact for overall survival (*χ* ^2^, 34.163; relative risk (RR), 2.766; 95% confidence interval (CI), 1.966–3.890; *P* < 0.001) and recurrence-free survival (*χ* ^2^, 25.616; RR, 2.727; 95% CI, 1.849–4.022; *P* < 0.001). Moreover, if biomarker risk score was taken into multivariate Cox regression analysis, rather than CD44, Shh, and Gli1 expression, biomarker risk score (*χ* ^2^, 11.744; RR, 1.999; 95% CI, 1.345–2.972; *P* = 0.001), and TNM stage were independent prognostic factors for overall survival, and biomarker risk score (*χ* ^2^, 7.183; RR, 1.848; 95% CI, 1.179–2.895; *P* = 0.007), TNM stage, and nodal classification were independent prognostic factors for recurrence-free survival in our study.

## 4. Discussion

The CD44 gene, located on chromosome 11p12-13, has various isoforms consisting of at least 19 exons. The CD44 protein is a class I transmembrane glycoprotein and is a major component of the extracellular matrix that regulates the function of cell-cell and cell-tissue adhesion. Moreover, the CD44 protein has been identified as a biomarker of side population cells [11] or cancer stem-like cells [12] in the gastric cell lines MKN-45, MKN-74, NCI-N87, and BGC-823. Hence, CD44 may be involved in several malignant biological processes, such as tumour initiation, development, and metastasis. As one of the most important signalling pathways, Shh has been implicated in the regulation of gastric cancer cell proliferation, migration, invasion, stem cell maintenance, and lymphangiogenesis. CD44 is required for Shh signalling pathway activation in various types of cancer, including ovarian [13], pancreatic [14], and prostate cancers [15]. Most studies have confirmed an interaction between CD44 and the Shh pathway* in vivo*. In contrast, Nanashima et al. [16] found no significant correlation between the expressions of Gli1 and CD44 in intrahepatic cholangiocarcinoma. There are very few studies in the literature evaluating the interaction between the Shh pathway and CD44 in gastric cancer cells. Song et al. [10] demonstrated that the Shh pathway was important for maintenance of cancer stem-like abilities in human gastric cancer cells. Yu et al. [17] found that overexpression of Shh signalling pathway genes was accompanied by an increase in CD44-positive cells in the MKN45 gastric cancer cell line. A similar result has been reported for breast cancer cells [18]. However, to the best of our knowledge, the correlation of CD44, Shh, and Gli1 in gastric cancer and their clinicopathological significance have not been reported in the literature. This is the first report revealing a positive relationship between CD44 expression and the levels of 2 important members of Hedgehog signalling pathway* in vivo*, suggesting that the interaction of CD44 and the Shh pathway may be involved in primary gastric cancer tumourigenesis, progression, and metastasis.

Most studies confirm that high CD44 [19], Shh [20], and Gli1 [21] expression is significantly associated with poorer clinicopathological parameters and worse overall survival in gastric cancer. It was worthy of note that most studies did not distinguish between patients who underwent radical resection and those receiving palliative surgery, which have significant differences in clinicopathological features and prognosis, when assessing the association of CD44, Shh, and Gli1 protein levels in GC. This study is the first to explore CD44, Shh, and Gli1 expression only in patients who underwent radical resection. Similar to studies that included both patients who underwent radical resection and palliative surgery, we also found an association between high CD44, Shh, and Gli1 expression and clinicopathological characteristics indicative of increased malignant potential, such as gross type, tumour differentiation, tumour invasion, and lymph node metastasis.

The clinical usefulness of CD44 expression to predict recurrence in GC is controversial. Hirata et al. [22] reported that expression of CD44 variant 9, an isoform of CD44, could predict recurrence in early gastric cancer. In contrast, Yong et al. found that the expression of CD44 was not associated with recurrence of gastric cancer [23]. The different proportion of patients receiving radical resection versus palliative surgery may have contributed to the different conclusions reached in these two studies. No previous studies have clarified the association between CD44 overexpression and tumour relapse or long-term survival only in gastric cancer patients who received radical resection. Moreover, this is the first study to demonstrate that patients with CD44-negative tumours have better overall survival and lower recurrence rate than patients with CD44-positive tumours after radical surgery. Similarly, it is also the first time to evaluate the overexpression of Shh and Gli1 proteins can predict worse survival outcome and early recurrence in gastric cancer.

To assess the aggressiveness of CD44, Shh, and Gli1 for gastric cancer, we established biomarker risk score system to evaluate the prognostic importance. The biomarker risk score system can discriminate survival differences of overall survival and recurrence-free survival and show the highest prognostic value from the multivariate Cox regression analysis result. This may partially explain why CD44 and Shh signaling pathway signatures are useful biomarkers for aggressive tumour behaviour in gastric cancer.

In summary, the cancer stem cell biomarker CD44 and Shh signaling pathway signatures can be used as novel diagnostic and therapeutic tools. It is necessary to further elucidate the mechanisms of aberrant Shh, Gli1 expression and the overexpression of CSCs markers in gastric cancer.

## Figures and Tables

**Table 1:** Clinicopathological characteristics of CD44, Shh, and Gli1 in gastric cancer after radical resection.

Factors	Cases	CD44			Shh			GLI1		
		Positive (*n* = 59)	Negative (*n* = 42)	*P* value	Positive (*n* = 72)	Negative (*n* = 29)	*P* value	Positive (*n* = 59)	Negative (*n* = 42)	*P* value
Age				0.089			0.777			0.936
<60 years	51	34 (57.6%)	17 (40.5%)		37 (51.4%)	14 (48.3%)		31 (50.8%)	20 (50.0%)	
≧60 years	50	25 (42.4%)	25 (59.5%)		35 (48.6%)	15 (51.7%)		30 (49.2%)	20 (50.0%)	
Gender				0.460			0.929			0.852
Male	62	38 (64.4%)	24 (57.1%)		44 (61.1%)	18 (62.1%)		37 (60.7%)	25 (62.5%)	
Female	39	21 (35.6%)	18 (42.9%)		28 (38.9%)	11 (37.9%)		24 (39.3%)	15 (37.5%)	
Tumor location				0.684			0.891			0.440
Upper 1/3	21	13 (22.0%)	8 (19.0%)		15 (20.8%)	6 (20.7%)		13 (21.3%)	8 (20.0%)	
Middle 1/3	25	13 (22.0%)	12 (28.6%)		19 (26.4%)	6 (20.7%)		12 (19.7%)	13 (32.5%)	
Lower 1/3	50	29 (49.2%)	21 (50.0%)		35 (48.6%)	15 (51.7%)		32 (52.5%)	18 (45.0%)	
Whole	5	4 (6.8%)	1 (2.4%)		3 (4.2%)	2 (6.9%)		4 (6.6%)	1 (2.5%)	
Tumor size				0.038			<0.001			<0.001
<5 cm	34	15 (25.4%)	19 (45.2%)		16 (22.2%)	18 (62.1%)		11 (18.0%)	23 (57.5%)	
≥5 cm	67	44 (74.6%)	23 (54.8%)		56 (77.8%)	11 (37.9%)		50 (82.0%)	17 (42.5%)	
Histological type				0.004			0.002			0.002
Differentiated	30	11 (18.6%)	19 (45.2%)		15 (20.8%)	15 (51.7%)		11 (18.0%)	19 (47.5%)	
Undifferentiated	71	48 (81.4%)	23 (54.8%)		57 (79.2%)	14 (48.3%)		50 (82.0%)	21 (52.5%)	
Gross type				0.501			<0.001			<0.001
Noninfiltration	30	16 (27.1%)	14 (33.3%)		13 (18.1%)	17 (58.6%)		10 (16.4%)	20 (50.0%)	
Infiltration	71	43 (72.9%)	28 (66.7%)		59 (81.9%)	12 (41.1%)		51 (83.6%)	20 (50.0%)	
T stage (7th)				<0.001			<0.001			<0.001
I	10	2 (3.4%)	8 (19.0%)		3 (4.2%)	7 (24.1%)		2 (3.3%)	8 (20.0%)	
II	21	7 (11.9%)	14 (33.3%)		7 (9.7%)	14 (48.3%)		3 (4.9%)	18 (45.0%)	
III	20	10 (16.9%)	10 (23.8%)		13 (18.1%)	7 (24.1%)		10 (16.4%)	10 (25.0%)	
IVa	30	23 (39.0%)	7 (16.7%)		29 (40.3%)	1 (3.4%)		26 (42.6%)	4 (10.0%)	
IVb	20	17 (28.8%)	3 (7.1%)		20 (27.8%)	0 (0.0%)		20 (32.8%)	0 (0.0%)	
N stage (7th)				0.005			0.010			<0.001
N_0_	39	15 (25.4%)	24 (57.1%)		21 (29.2%)	18 (62.1%)		14 (23.0%)	25 (62.5%)	
N_1_	16	9 (15.3%)	7 (16.7%)		11 (15.3%)	5 (17.2%)		11 (18.0%)	5 (12.5%)	
N_2_	24	17 (28.8%)	7 (16.7%)		21 (29.2%)	3 (10.3%)		16 (26.2%)	8 (20.0%)	
N_3_	22	18 (30.5%)	4 (9.5%)		19 (26.4%)	3 (10.3%)		20 (32.8%)	2 (5.0%)	
TNM stage (7th)				<0.001			<0.001			<0.001
I	22	4 (6.8%)	18 (42.9%)		5 (6.9%)	17 (58.6%)		2 (3.3%)	20 (50.0%)	
II	21	10 (16.9%)	11 (26.2%)		15 (20.8%)	6 (20.7%)		9 (14.8%)	12 (30.0%)	
III	33	45 (76.3%)	13 (31.0%)		52 (72.2%)	6 (20.7%)		50 (82.0%)	8 (20.0%)	

**Table 2:** Univariate and multivariate analysis for overall survival in gastric cancer after radical resection.

Factors	Univariate regression analysis				Multivariate regression analysis			
	*χ* ^2^ value	OR	95% CI	*P* value	*χ* ^2^ value	OR	95% CI	*P* value
Age	0.001	0.995	0.589–1.679	0.984				
Gender	0.076	0.927	0.540–1.592	0.783				
Tumor location	0.052	0.966	0.713–1.307	0.820				
Tumor size	10.301	2.957	1.525–5.733	0.001				
Histological type	7.097	2.457	1.268–4.760	0.008				
Gross type	5.811	2.252	1.164–4.357	0.016				
T stage	11.943	3.370	1.692–6.713	0.001				
N stage	5.334	2.473	1.147–5.333	0.021				
TNM stage	21.978	3.070	1.921–4.906	<0.001	11.856	1.346	1.137–1.594	0.001
CD44 expression	16.049	3.589	1.921–6.706	<0.001				
Shh expression	13.707	4.490	2.028–9.945	<0.001				
Gli1 expression	28.800	8.927	4.013–19.858	<0.001	9.970	4.247	1.731–10.423	0.002

**Table 3:** Univariate and multivariate analysis for disease-free survival in gastric cancer after radical resection.

Factors	Univariate regression analysis				Multivariate regression analysis			
	*χ* ^2^ value	OR	95% CI	*P* value	*χ* ^2^ value	OR	95% CI	*P* value
Age	0.461	—	—	0.497				
Gender	0.323	—	—	0.570				
Tumor location	0.706	—	—	0.401				
Tumor size	8.632	3.187	1.471–6.904	0.003				
Histological type	2.391	—	—	0.122				
Gross type	4.816	2.372	1.097–5.130	0.028				
T stage	20.271	1.912	1.442–2.535	<0.001				
N stage	20.429	1.841	1.413–2.398	<0.001	4.368	1.334	1.018–1.747	0.037
TNM stage	27.076	2.663	1.841–3.851	<0.001	7.473	1.940	1.206–3.121	0.006
CD44 expression	11.981	3.545	1.731–7.258	0.001				
Shh expression	10.853	4.836	1.893–12.352	0.001				
Gli1 expression	21.233	7.806	3.257–18.707	<0.001	6.387	3.403	1.316–8.796	0.011
